# Hydration of Carboxyl Groups: A Route toward Molecular Recognition?

**DOI:** 10.1021/acs.jpcb.0c03609

**Published:** 2020-04-30

**Authors:** Michael Di Gioacchino, Fabio Bruni, Silvia Imberti, Maria Antonietta Ricci

**Affiliations:** † Dipartimento di Scienze, Universitá degli Studi Roma Tre, via della Vasca Navale 84, 00146 Roma, Italy; ‡ UKRI-STFC, ISIS Neutron and Muon Source, Rutherford Appleton Laboratory, Harwell Campus, Chilton, Didcot OX11 0QX, U.K.

## Abstract

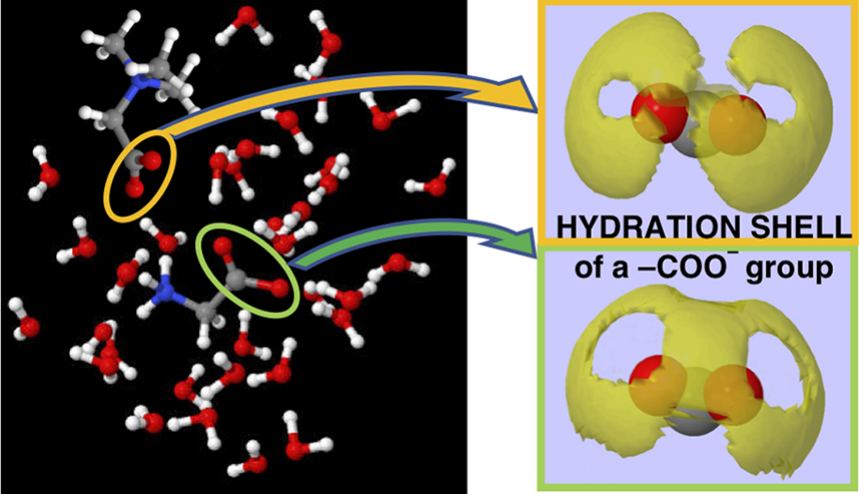

On Earth, water plays an active role in cellular life, over several scales of distance and time. At a nanoscale, water drives macromolecular conformation through hydrophobic forces and at short times acts as a proton donor/acceptor providing charge carriers for signal transmission. At longer times and larger distances, water controls osmosis, transport, and protein mobility. Neutron diffraction experiments augmented by computer simulation, show that the three-dimensional shape of the hydration shell of carboxyl and carboxylate groups belonging to different molecules is characteristic of each molecule. Different hydration shells identify and distinguish specific sites with the same chemical structure. This experimental evidence suggests an active role of water also in controlling, modulating, and mediating chemical reactions involving carboxyl and carboxylate groups.

## Introduction

Recent views of water as a “life solvent” suggest that it actively engages and interacts with biomolecules in subtle and complex ways, via delicate interplay and feedback mechanisms. Biomolecules and their aqueous environment cannot be regarded as distinct entities.^1^ While molecules, as for instance proteins, shape the shell of water that surrounds them and influence its dynamics, the structure and dynamics of this hydration shell seem to feed back onto those aspects of the protein structure and dynamics that are essential for their function.^1−4^ The hydration shell preserves the native structure of proteins, sometimes mediates the interaction with receptors and substrates, makes protons available for signal transmission, and promotes protein vibrational dynamics.^5^

When two organic molecules encounter each other, the first step of their interaction is a recognition event through weak intermolecular forces, eventually leading to a chemical reaction and/or formation of complexes. The extent of the role of water in molecular recognition is still unknown.^2,5−7^ However, it is agreed that complexation in water is driven by a balance between entropic and enthalpic gains, where the cost of moving water from the hydration shell of a molecule into the bulk solvent is not negligible.^2,7^ The energy cost of removing water from the hydration shell of solutes (being a receptor, a protein, or a ligand) clearly depends on the strength of their interaction and in particular on the number of H-bonds between water molecules and the solvent. Thus, atomic-level knowledge of the solute hydration shells and the spatial arrangement of water molecules around the solute may unveil the route toward a deeper understanding of the recognition events and a better drug-design strategy.

Investigations of the hydration of protein surfaces is usually performed by cryogenic X-ray crystallography.^8^ Here, instead of looking at an entire macromolecule in the crystalline phase at low temperatures, we propose to study at room temperature the hydration shell of specific groups (the carboxyl or carboxylate group in the present instance), belonging to different molecules, in solution. This will eventually help to establish common features or single out differences in their interaction with water, if any. This is done by neutron total scattering experiments, exploiting the isotopic H/D substitution, interpreted by means of a computer simulation refinement of the measured differential cross sections.

## Methods

Neutrons are a particularly suitable probe to investigate the structure of aqueous solutions because they are strongly scattered by hydrogen atoms and are able to distinguish between hydrogen and deuterium isotopes^9^ so that the diffraction pattern from the solutions with different H/D proportions can be markedly different. Consequently, the structural information available increases with the number of isotopically substituted solutions exposed to the neutron beam. Moreover, the availability of the coordination of water hydrogens relative to the solute atomic sites provides information on the relative orientation of water and solute, missing in the X-ray diffraction experiment. Here, we report the results of a series of total neutron scattering experiments on aqueous solutions of small biomolecules, performed by exploiting the H/D isotopic substitution and interpreted with the computational modeling technique called “Empirical Potential Structural Refinement” (EPSR).^10^ The result is a description of the hydration shell at the atomic scale. All of the molecules considered have a carboxyl or carboxylate group, and we will discuss here only the hydration of these groups, leaving the discussion of the hydration of the entire molecules and their influence on the water network to more extensive reports in future and previous publications.^11−13^ In detail, we refer to aqueous solutions, at ambient conditions, of betaine, ectoine,^12^ glycine,^13^ and trigonelline, in comparison with the previously published solutions of glutathione.^11^

### Experimental Section

The experiments have been performed at the SANDALS diffractometer, installed at the ISIS Neutron and Muon source (Rutherford Appleton Laboratory, STFC, U.K.), by exploiting the H/D isotopic substitution^14,15^ on aqueous solutions of betaine, ectoine,^12^ glycine (all three solutes at a concentration of 1 solute mole per 30 water moles), and trigonelline (at a concentration of 1 solute mole per 50 water moles). Previous experiment on a solution of glutathione^11^ was performed at a concentration of 1 solute mole per 130 water moles. The quoted different concentrations do not affect the results of our analysis, as we are only interested in the first hydration shell of the solutes and, in all cases, the number of water molecules per solute is much higher than the number of molecules in this shell.

The measured quantity is the total interference differential scattering cross section, which is a linear combination of the Fourier transforms of all pair radial distribution functions (RDFs), namely, the partial structure factors *S*_αβ_, weighed according to the concentrations, *c*_α_, *c*_β_, and neutron scattering lengths, *b*_α_ and *b*_β_,^9^ of the pair components
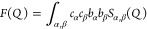
1where *Q* is the modulus of the exchanged momentum in the scattering event.

Information on the hydration of the carboxyl/carboxylate groups is given by the partial structure factors relative to the pairs formed by the carboxyl/carboxylate oxygens (O) and the water atoms (Ow, Hw) and their Fourier transforms. This information cannot be directly extracted from the experimental data, since the number of measured *F*(*Q*)s in practice cannot equal the number of atomic pairs.^15^ As a consequence, the data are modeled and interpreted using the Monte Carlo simulation code described in the next section.

### EPSR Fitting

**Figure 1:**
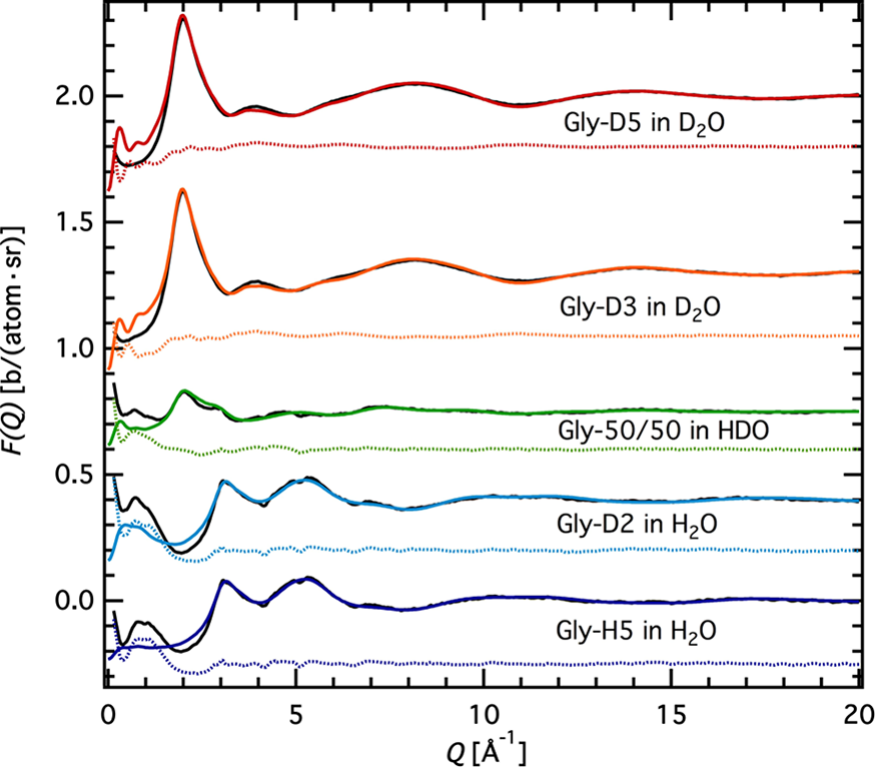
*F*(*Q*)s of the 1:30 glycine–water solution at room temperature: data (solid black lines), fits (solid colored lines), and residuals (down-shifted, dashed colored lines). Gly-D5 labels the fully deuterated glycine molecule; Gly-D3 labels the glycine molecule with deuterated amino group; Gly-50/50 is 50% mixture of natural and fully deuterated glycine; Gly-D2 labels the glycine molecule with deuterated methylene group; and Gly-H5 labels natural glycine. Data have been shifted for clarity.

The Empirical Potential Structure Refinement Code (EPSR)^10^ has been so far successfully employed to investigate the interaction of small molecules with water.^16−18^ It is a Monte Carlo routine, which refines an interaction potential and a real space structural model of the sample against the experimental data, starting from a seed potential model and a random distribution of molecules in the simulation box. Provided that the simulation box has the same composition and density as those of the real sample, the more isotopic substituted samples are available, the better constraint is imposed to the fitting code, and the better reliability of the atomic structure of the sample is achieved. Importantly, the seed potential model, usually a Lennard-Jones potential plus fractional charges, must be suited to represent the real sample. In the present case, the interaction models for the different solutes have been adapted from refs (19, 20). The simple point charge/extended (SPC/E) model^21^ has been used to describe water interactions. After equilibrating on the seeding potential for over about 10^3^ iterations, we started the potential “refinement” loop. During this phase of the data analysis, the algorithm iteratively adds a numerical correction to the analytical seeding potential that guides the configuration toward an improved agreement with the data. Once the fit cannot improve further, the production run can start, with statistics accumulated over at least 10^4^ configurations. An example of the data fit obtained by the EPSR simulation is shown in Figure 1 (all other fits are of the same quality).

**Figure 2:**
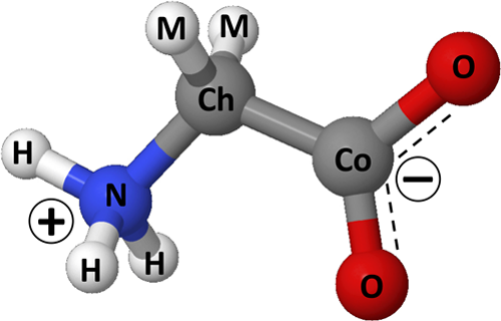
Atom labeling for the glycine molecule used in the EPSR analysis and in the pair distribution functions reported above. The carbon atoms are labeled as Ch and Co to evidence their different environments. N and H label the amide site components, and the oxygens of carboxylic group are labeled O. All hydrogens bonded to carbon sites are labeled M.

**Figure 3:**
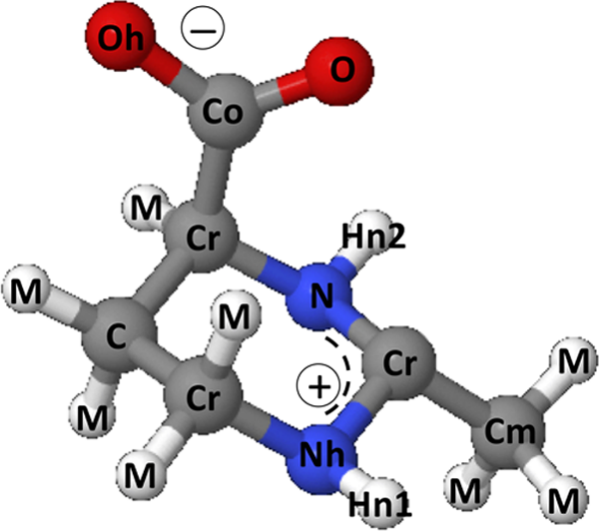
Structure of the ectoine molecule and atom labeling used in the EPSR analysis and in the pair distribution functions reported above. The −COO^–^ group is labeled Co for carbon and O and Oh for the oxygens. The carbon atoms on the ring are labeled C. The nitrogen atoms are respectively labeled N and Nh, and the associated hydrogen atoms are labeled Hn2 and Hn1, respectively. The carbon atom of the methyl group is labeled Cm. Finally, all hydrogens bounded to carbon sites are labeled M.

**Figure 4:**
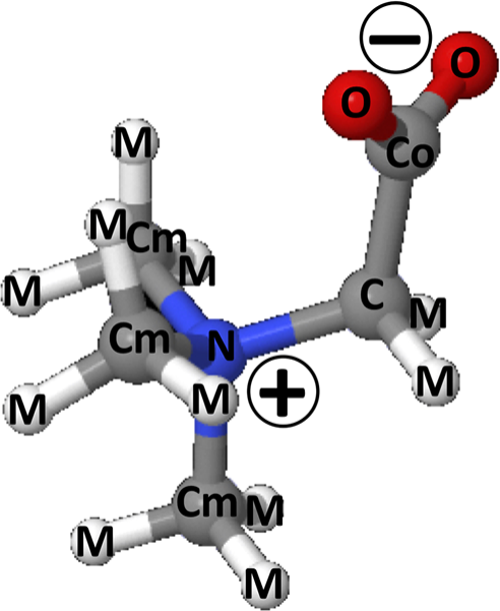
Structure of the betaine molecule and atom labeling used in the EPSR analysis and in the pair distribution functions reported above. The carbon atoms are labeled C, Co, and Cm to evidence their different environments; the nitrogen atom is labeled N, and the oxygens of carboxylic group are labeled O. All hydrogens bonded to carbon sites are labeled M.

**Figure 5:**
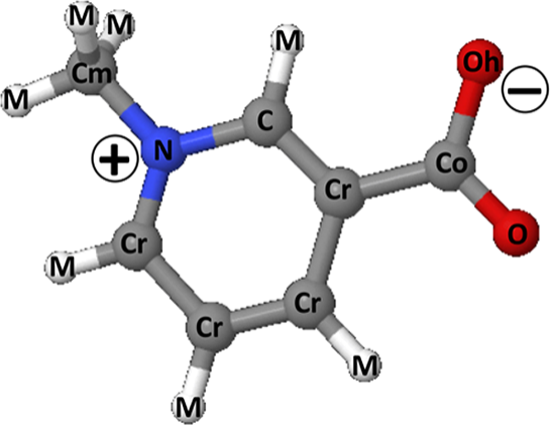
Structure of the trigonelline molecule and atom labeling used in the EPSR analysis and in the pair distribution functions reported above. The −COO^–^ group is labeled Co for carbon and O and Oh for the oxygens. The carbon atoms on the ring are labeled Cr and C, where C is considered the center atom of the molecule. The nitrogen atom is labeled N. The carbon atom of the methyl group is labeled Cm. Finally, all hydrogens bounded to carbon sites are labeled M.

All of the information on the EPSR box used for the analysis of the gluthatione aqueous solution is reported in ref (11). The atomic labels and potential parameters^19,20^ adopted for all the other solutes are reported in Tables 1–4 and Figures 2–5.

**Table 1:** Reference Potential Parameters Used in the EPSR Simulation of Glycine^a^

atom label	ϵ (kJ/mol)	σ (Å)	*q* (e)
N	0.71128	3.25	–0.30
Ch	0.41420	3.80	0.21
Co	0.43932	3.75	0.70
O	0.87864	2.96	–0.80
H	0	0	0.33
M	0	0	0

a Atoms are labeled according to Figure 2. The box contains 30 glycine molecules and 900 water molecules.

**Table 2:** Reference Potential Parameters Used in the EPSR Simulation of Ectoine^a^

atom label	ϵ (kJ/mol)	σ (Å)	*q* (e)
Oh	0.71128	3.00	–0.58
Oc	0.87864	2.96	–0.50
N	0.83700	3.70	–0.70
Nh	0.83700	3.70	–0.70
Co	0.43932	3.75	0.55
C	0.31400	3.80	0.27
Cm	0.08370	4.55	0.05
Hn1	0	0	0.575
Hn2	0	0	0.225
Mc	0	0	0

a Atoms are labeled according to Figure 3. The box contains 30 ectoine molecules and 900 water molecules.

**Table 3:** Reference Potential Parameters Used in the EPSR Simulation of Betaine^a^

atom label	ϵ (kJ/mol)	σ (Å)	*q* (e)
N	0.71128	3.25	–0.30
C	0.41420	3.80	0.60
Cm	0.71145	3.80	0.20
Co	0.43932	3.75	0.70
O	0.87864	2.96	–0.80
M	0	0	0

a Atoms are labeled according to Figure 4. The box contains 30 betaine molecules and 900 water molecules.

**Table 4:** Reference Potential Parameters Used in the EPSR Simulation of Trigonelline^a^

atom label	ϵ (kJ/mol)	σ (Å)	*q* (e)
Oh	0.71128	3.00	–0.58
O	0.87864	2.96	–0.50
Co	0.43932	3.75	0.55
Cr	0.31400	3.80	–0.146
C	0.31400	3.80	–0.146
Cm	0.08370	4.55	0.36
N	0.83700	4.55	0.90
M	0	0	0

a Atoms are labeled according to Figure 5. The box contains 60 trigonelline molecules, 60 Cl^–^ atoms, 60 H^+^, and 3000 water molecules.

In the case of betaine and glycine,^13^ the simulation was performed without distinguishing the two oxygen sites on the carboxyl group, based on a preliminary test (data not shown).

The output of the EPSR fitting procedure is a collection of molecular configurations, compatible with the experimental data. From these configurations, the structural quantities of interest can be evaluated. Here, we report the pair radial distribution functions (RDFs) between the carboxyl/carboxylate oxygens (O) and the water atoms (Ow, Hw), the distribution functions of the angles defined by a selected triplet of atoms, and the three-dimensional distribution of water molecules around a specific site through the spatial distribution function (SDF) defined in refs.^22,23^

## Results and Discussion

**Figure 6:**
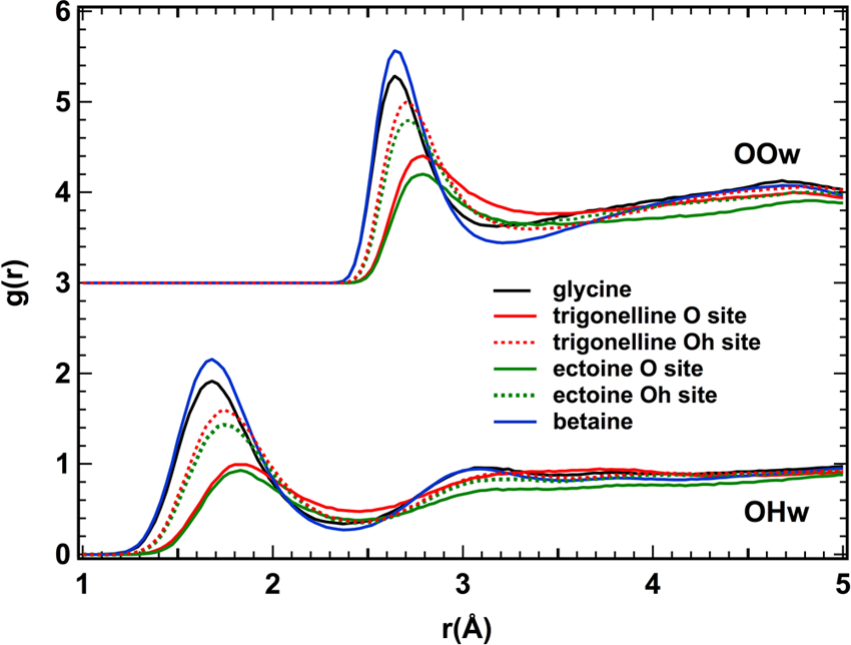
Radial distribution functions (RDF) of water sites (Ow,Hw) around the O sites of the carboxyl groups of glycine (black), trigonelline (red), ectoine (green), and betaine (blue) in solution. These functions represent the probability density that given a carboxyl O atom at the origin of the reference frame, an Ow or Hw atom sits at a distance *r*. The two oxygen sites of trigonelline and ectoine are labeled O (double bonded to the C site, solid line) and Oh (single bonded to the C site, dashed line). Oh has a larger negative charge than O, according to the optimized potential for liquid simulation (OPLS) potential model for acids;^20^ the Oh sites of both trigonelline and ectoine bind water closely than the O site. The OPLS potential for glycine and betaine does not distinguish the two O sites.^19^ Both the OOw and OHw RDFs of the glycine and betaine solutions have the first neighbor peak at shorter distances compared to the case of trigonelline and ectoine.

The RDFs shown in Figure 6 represent the density of probability that, given an O atom of the solute (glycine, betaine, trigonelline, and ectoine), carboxyl, or carboxylate group at the origin of the reference frame, an Ow or Hw atom can be found at distance *r* from it.

In all samples, the carboxyl groups form hydrogen bonds (HBs) with water. This is witnessed by the first neighbor peak of the OHw RDF at ∼1.7–1.8 Å and the first peak of the OOw RDF between 2.7 and 2.8 Å. Indeed, similar peak positions are found in pure water for OwHw and OwOw, respectively, as signatures of hydrogen bonding. Nevertheless, we notice that the investigated amino acid, namely, glycine, and derivative amino acid, namely, betaine, form the shortest and thus stronger bonds with the first neighbour water molecules, compared with the other two solutes (trigonelline and ectoine). In the latter solutions, where we distinguish the two oxygens of the carboxyl site, the doubly bonded oxygen forms the longest and weakest bonds. Importantly, the intensity and width of the peaks shown in Figure 6 depends on the parent molecule. Nevertheless, according to the EPSR simulation, the number of water molecules within the hydration shell (data not shown) does not sensibly differ, at odds with previous spectroscopic evidence.^24^ Different hydration structures for carboxyl groups have been found also by molecular dynamic simulations.^25^

**Figure 7:**
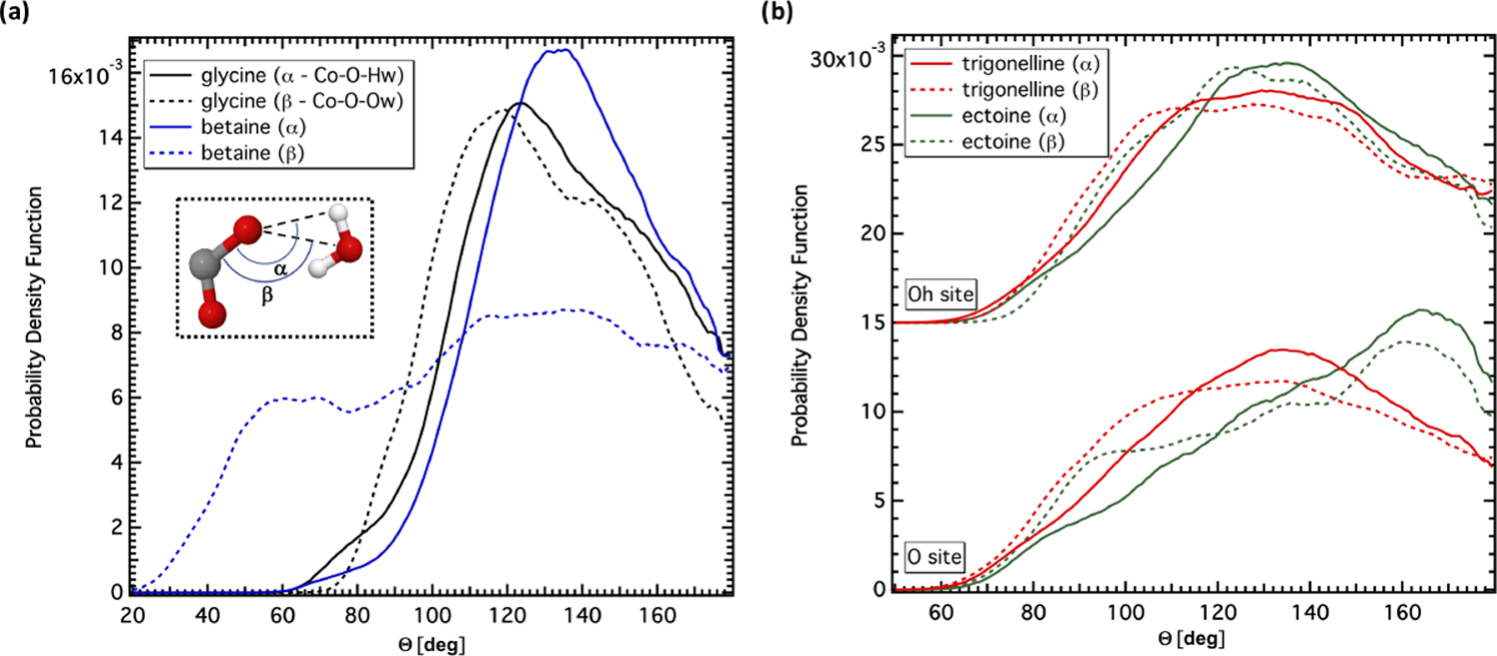
Distribution functions of the α =  (solid) and β =  (dashed) angles (see inset for definition). (a) Distribution functions for glycine (black) and betaine (blue). (b) Distribution functions for trigonelline (red) and ectoine (green) at the O and Oh sites (vertically offset for clarity).

In addition to the strength of the HBs, also the orientational arrangement of the water molecules within the individual carboxyl groups hydration shell is different. Indeed, the distribution functions of the angles defined by the intramolecular C–O bond (notice that the label of the carbon atom in question in the EPSR simulation box is Co, according to Figures 2–5 and Tables 1–4) and the water atoms, namely, α =  and β =  (see Figure 7) evidence additional differences among the four investigated hydration shells. In the case of glycine, both α and β angles have quite sharp bimodal distribution, with a main peak and a shoulder. The α distribution has the main peak at ∼120° and the shoulder at ∼150°; the β distribution is down-shifted by ∼10°. It is tempting to ascribe the observed two contributions to the two C–O bonds of the carboxyl group. The α distribution for betaine is sharper and centered at ∼130°, while the β distribution is almost flat between 50 and 180°. In the case of trigonelline and ectoine, the distribution functions of the α and β angles formed at the Oh site are very similar to each other, broad and centered at ∼130°, implying linearity of the  angle. Interestingly, the angle distribution functions at the other site on the carboxyl group of trigonelline, namely, the O site, is similar to that of the Oh site. On the contrary, the distribution function at the O site of the ectoine shows a maximum at ∼180° for both α and β angles, suggesting the predominance of linear HBs. The observed difference between the hydration of the two C–O sites of ectoine supports previous assignment of the double structure of the glycine distribution functions to the individual C–O bonds. Finally, we notice that the distribution functions of all α angles show a structure at ∼75–80° (less visible in Figure 7b, due to the scale of the vertical axis): this is likely because water molecules are H-bonded to the companion C–O site.

To get better insight into the different hydration shells of the four investigated molecules, we have determined within the EPSR code the three-dimensional shape of the carboxyl hydration shells. This is done by calculating the SDF from the recorded molecular configurations. These functions allow to evidence the region of space where the probability of finding a water molecule within a given distance from the atom at the origin of the reference frame exceeds a threshold value.

**Figure 8:**
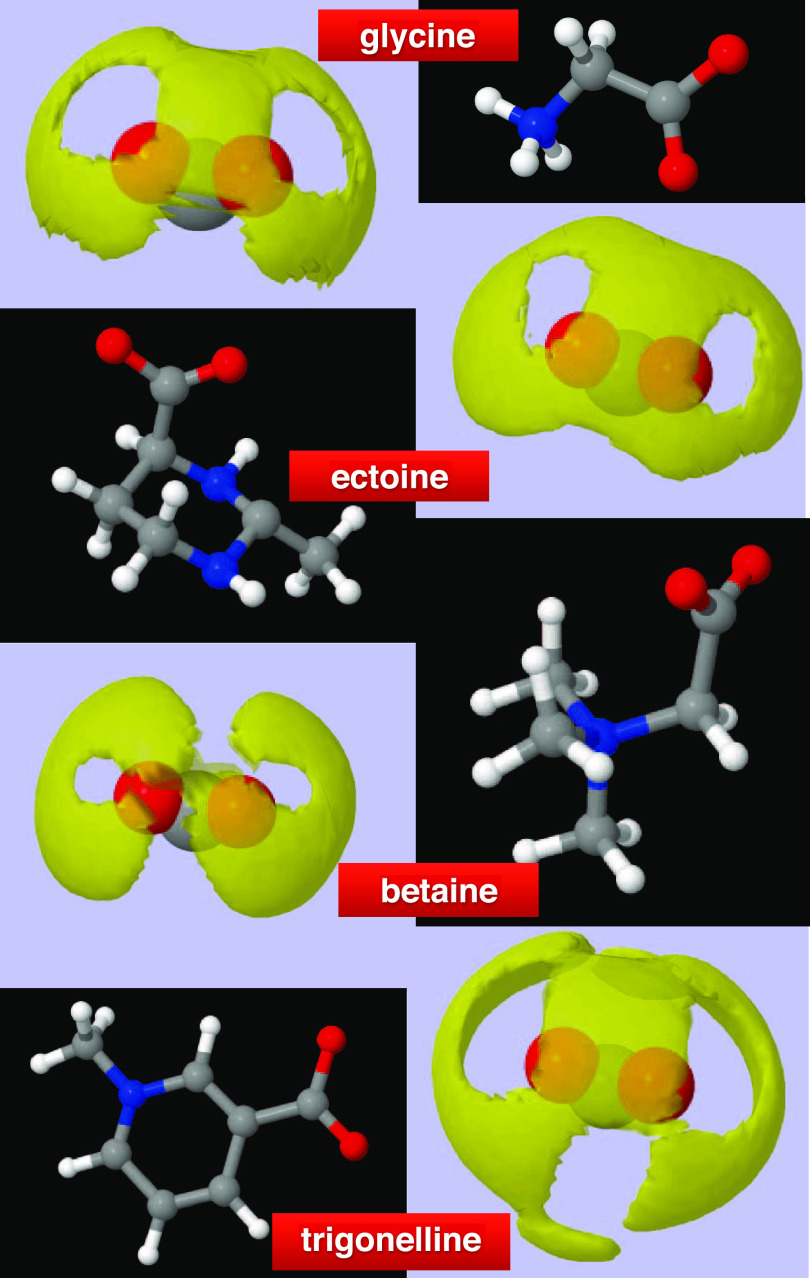
Ball-and-stick structure of glycine, ectoine, betaine, and trigonelline and spatial density functions (SDFs) for water around the carboxyl (−COO^–^) groups of these molecules in solution. The yellow surface contours enclose the highest-density regions that contain 35% of the water molecules within the first neighboring shell of the carbon atom of the carboxyl group of each molecule. This corresponds to a cutoff distance of 4.26 Å for glycine and betaine, 4.40 Å for trigonelline, and 4.50 Å for ectoine. Carbon is represented in gray and oxygen in red. The different shapes of the hydration shell, as evidenced by the SDF, identify and distinguish each molecule exposing a COO^–^ site to the solvent.

Figure 8 shows the SDFs of water around the carboxyl groups of glycine, ectoine, betaine, and trigonelline, whose ball-and-stick molecular structure is also shown in the figure. The SDFs give a pictorial view of the hydration shells of these groups and evidence markedly different shapes and extensions. In particular, we notice that the hydration shell of betaine markedly differs from those of others: it is the tightest one, with a unique symmetry, indicating a very low probability to have water molecules between the two oxygens of the carboxyl group. As a matter of fact, this molecule forms the strongest HB, with a sharper α distribution and a flatter β distribution (see Figure 7), due to the large probability density at low θ values of the β angle. The hydration shell of ectoine looks flatter compared to those of the others: this is likely due to the presence of molecules forming β angles at about 180°. At this stage, it is reasonable to state that each hydration shell identifies a different molecule.

**Figure 9:**
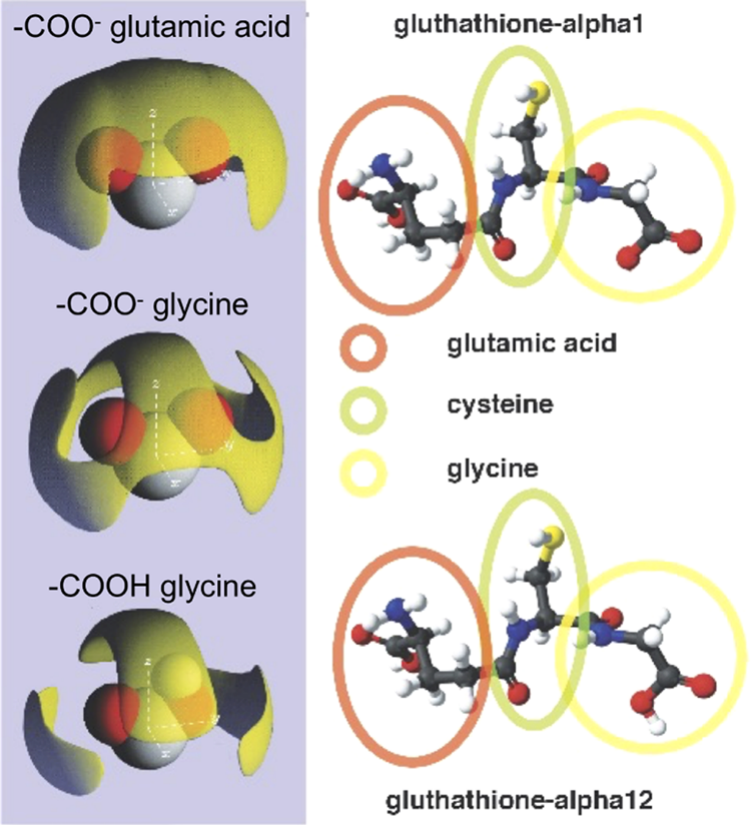
Ball-and-stick structure of the two anomers (α1 and α12) of glutathione and spatial density functions (SDFs) for water around carboxyl (−COO^–^) and carboxylate (−COOH) groups of glutathione in solution. The red, green, and yellow circles depict the glutamic acid and cysteine and glycine amino acids, composing glutathione, respectively. Panels on the right (redrawn from ref (11)) show the spatial density functions (SDFs) for water around the carboxyl groups of the glutamic acid (top), glycine acid (middle), and glycine carboxylate group (bottom). As in Figure 8, the yellow surface contours enclose the top 35% of the water molecules within the first neighboring shell distance from the carbon atom (4.26 Å).

Present results are corroborated by previous ones obtained for the small peptide gluthathione aqueous solutions,^11^ redrawn in Figure 9. In that case, the different hydration shells of the two carboxyl groups, one belonging to the glutamic acid and the other to the glycine end of the molecule, introduce an asymmetry in the hydration of the α1 glutathione anomer, not otherwise obvious, given the two identical carboxyl ends. Notice that the SDF around the glutamic acid end is different from all those reported in Figure 8, and more interestingly, the glycine end of the glutathione α1 anomer has a different hydration compared to the glycine molecule. Less surprising, although relevant, is the difference between the hydration shells of the carboxilate group of glutathione-α12 anomer and that of the carboxyl groups. Thus, the shape of the hydration shell not only allows to distinguish between the two anomers but also defines a “head” and a “tail” within the same molecule. This might play a role in the early stages of protein folding and protein–ligand interactions.

## Conclusions

Our experiments evidence remarkable differences between the hydration shells of carboxyl and carboxylate groups belonging to different molecules. These pertain to the strength and linearity of the HBs and to the orientation and spatial occupancy of water molecules relative to the the bisector of the  angle. An obvious consequence of these findings is that the energy cost of removing a water molecule from each COO^–^ hydration shell differs depending on the whole individual molecule. In this respect, our findings may represent the microscopic counterpart of the thermodynamic binding studies.^2^ We can argue that the hydration shell of the carboxyl group is the fingerprint of the individual molecules. At this stage, we can only speculate whether these differences depend either on the steric hindrances of the molecule, or on their different electronic charge distributions, or both of them. Nevertheless, our findings could be another example of the active role of water as a solvent, as by shaping the hydration shell of the carboxyl groups, it and can control and mediate the recognition events during chemical reactions in solution.
